# Supplementary figures and images for: In Search of Disentanglement in Tandem Mass Spectrometry Datasets
Source: Biomolecules. 2023 Sep 4;13(9):1343. doi: 10.3390/biom13091343 (PMC10526774; doi:10.3390/biom13091343)

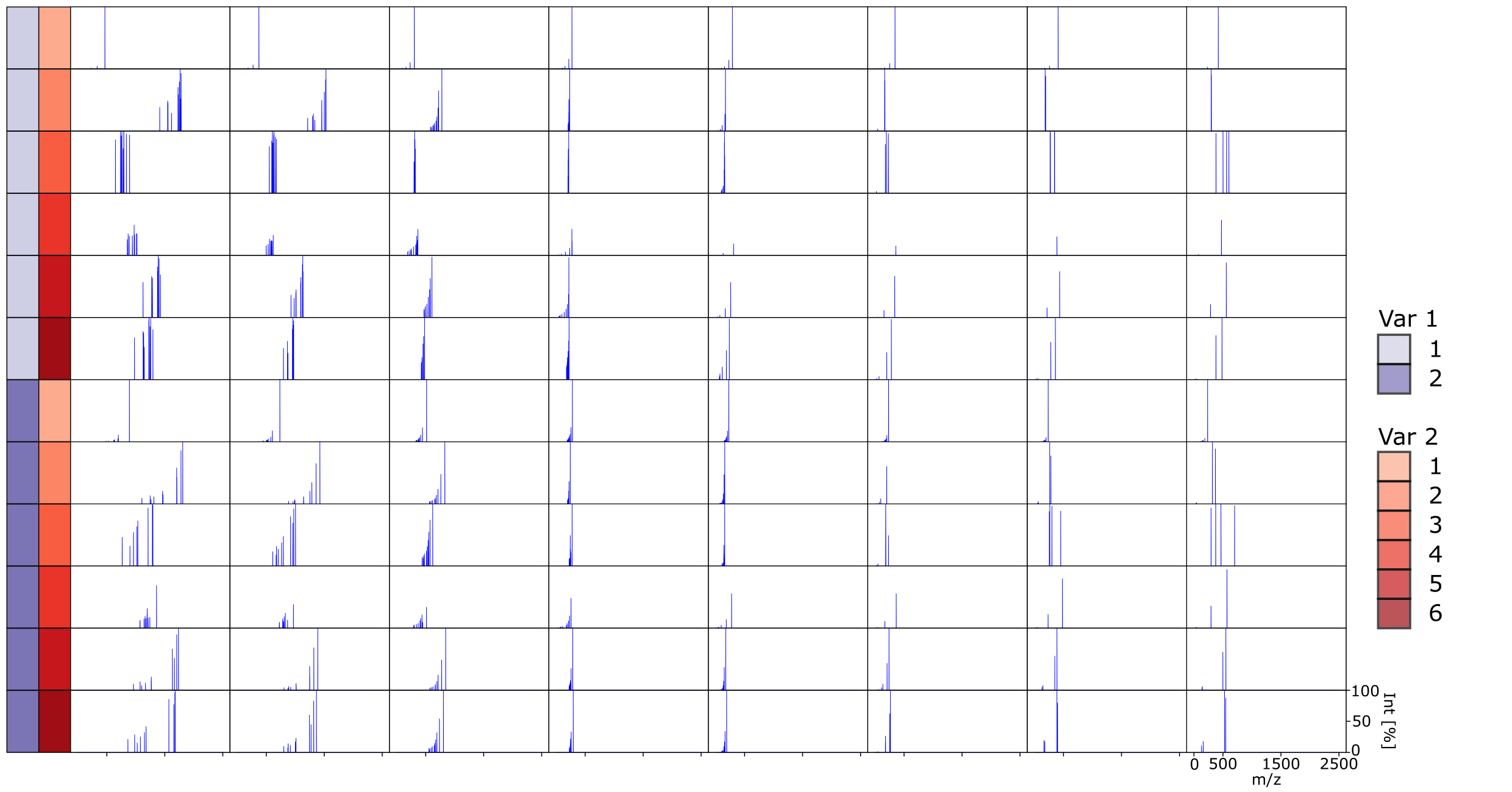

Supplement: Supplementary file 1 [file biomolecules-13-01343-s001.zip › Figure_S1.png]

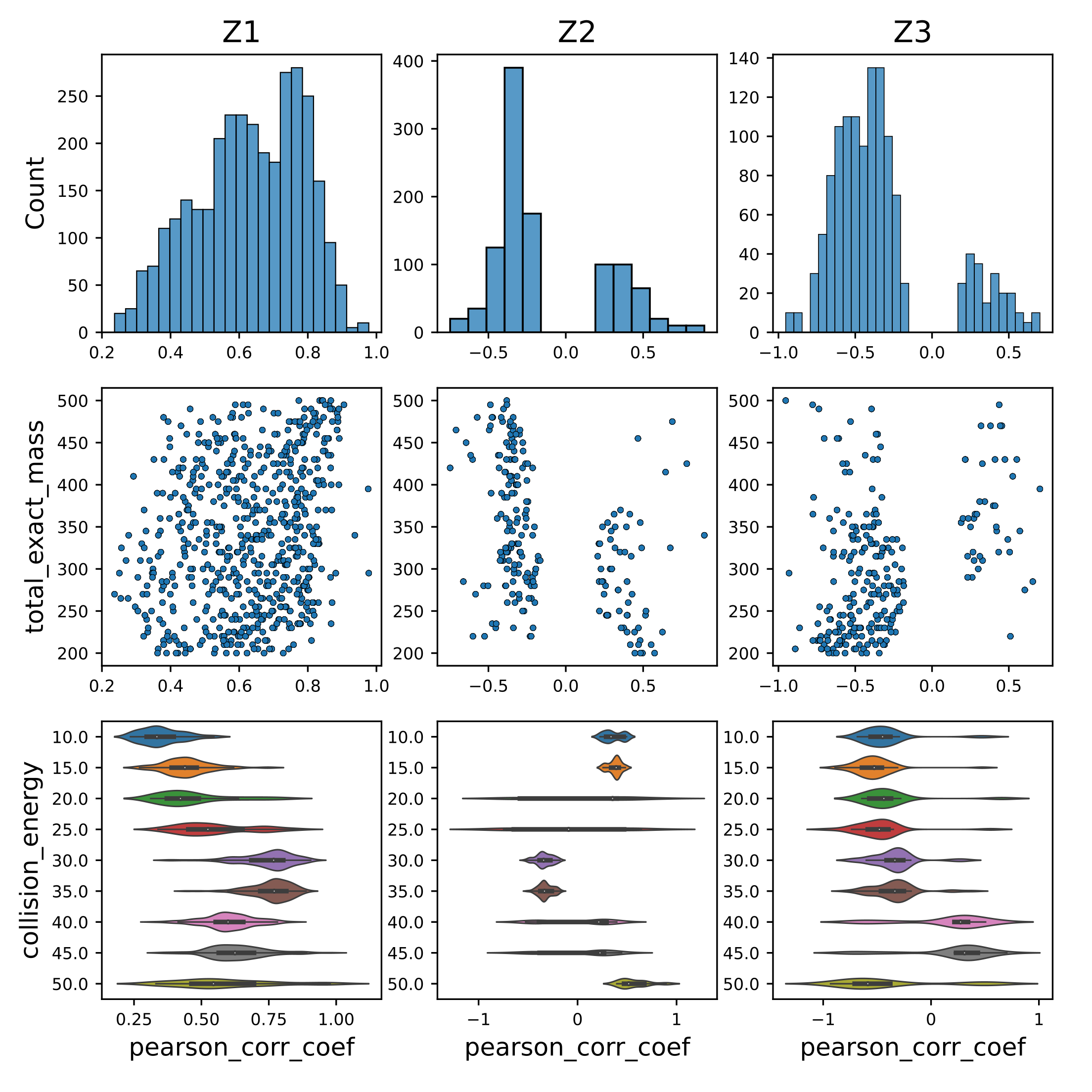

Supplement: Supplementary file 1 [file biomolecules-13-01343-s001.zip › Figure_S2.png]

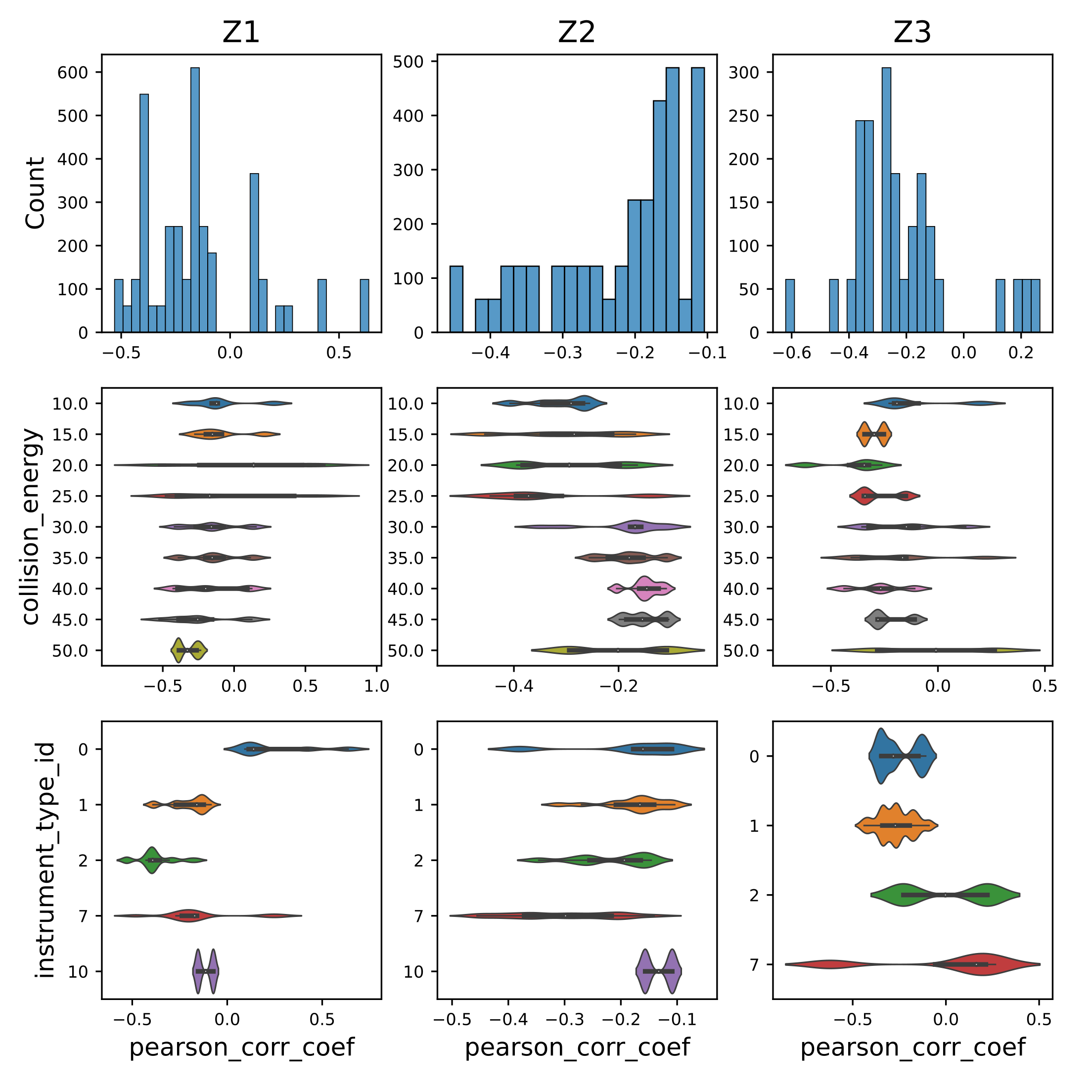

Supplement: Supplementary file 1 [file biomolecules-13-01343-s001.zip › Figure_S3.png]

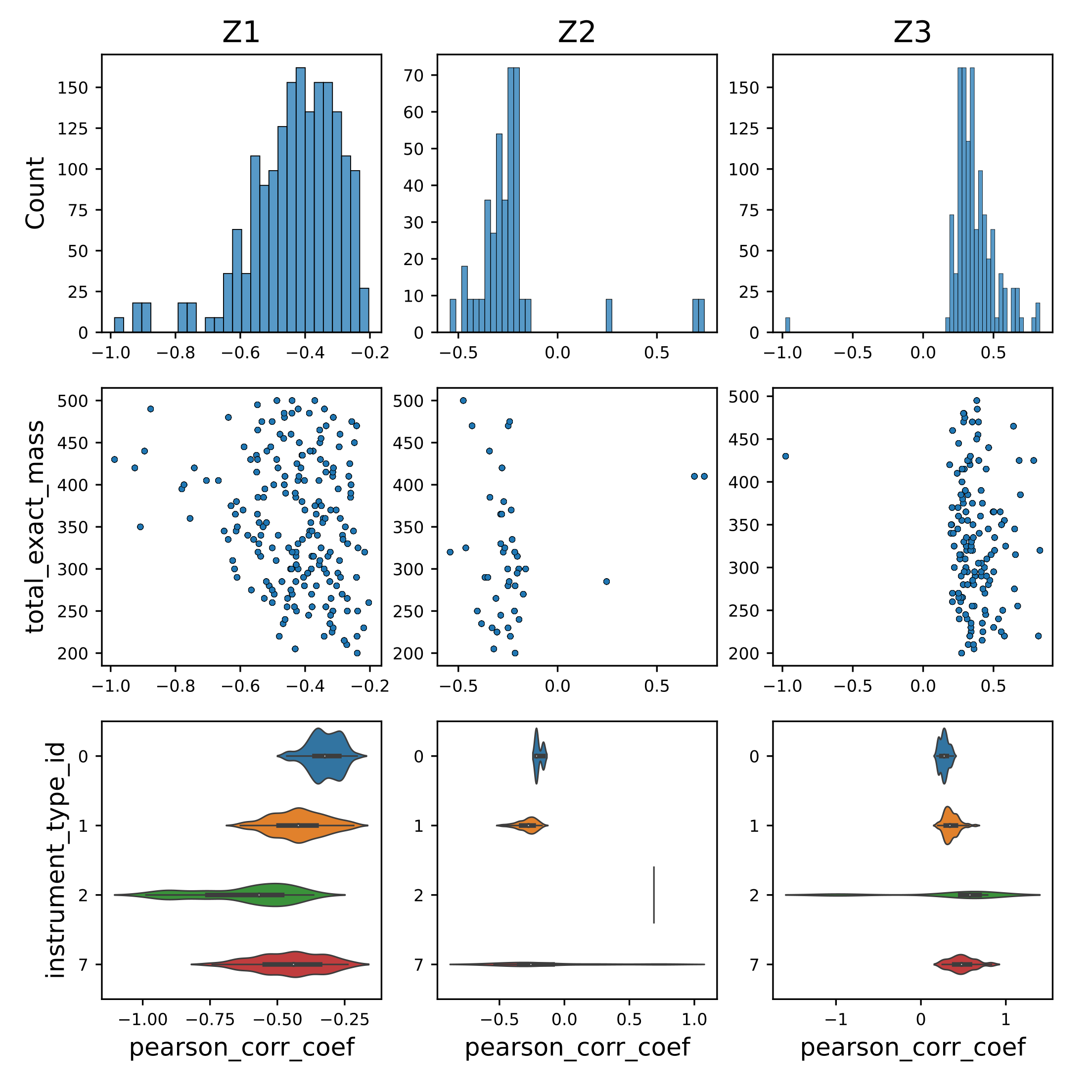

Supplement: Supplementary file 1 [file biomolecules-13-01343-s001.zip › Figure_S4.png]

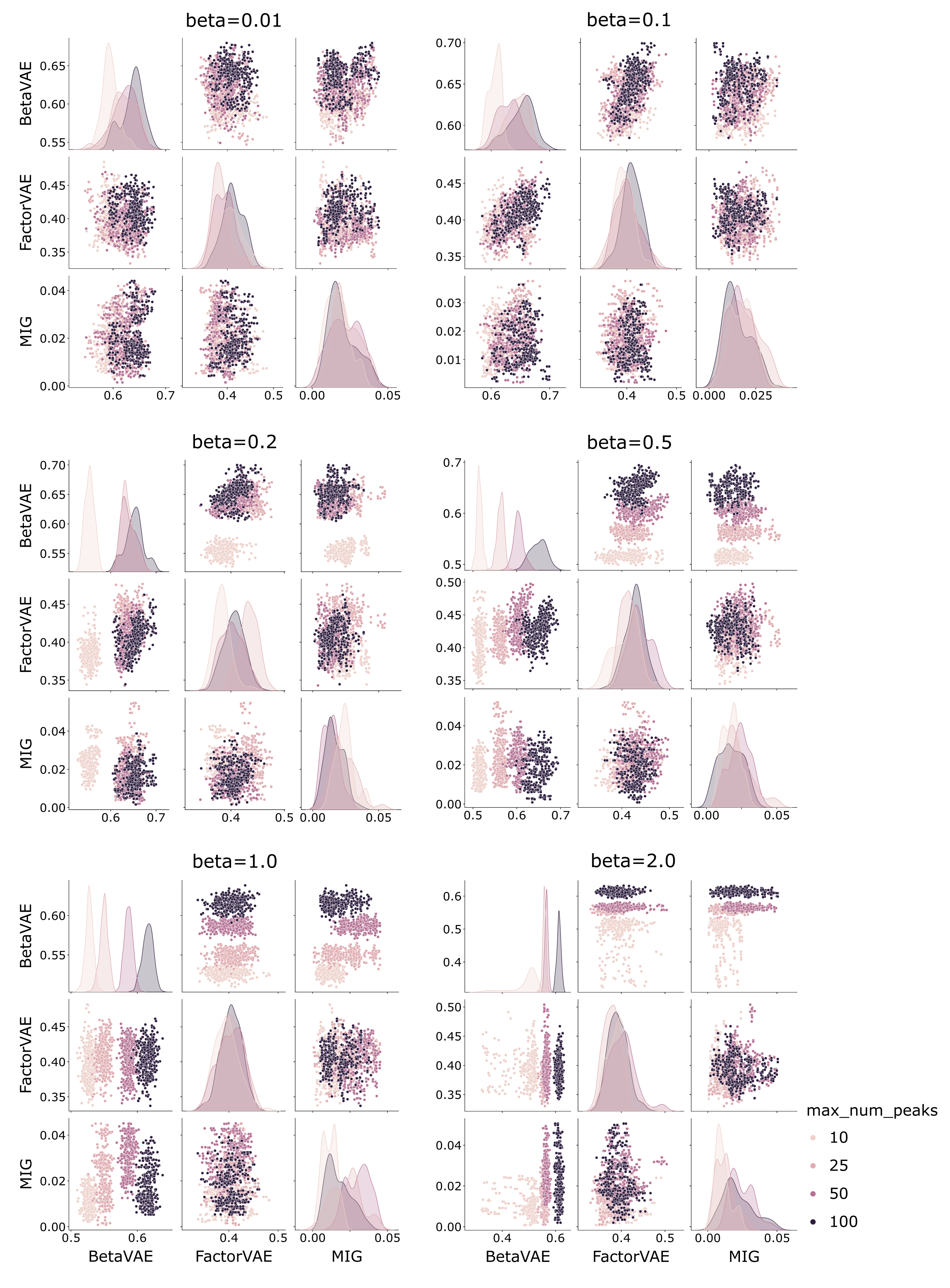

Supplement: Supplementary file 1 [file biomolecules-13-01343-s001.zip › Figure_S5.png]
